# ZFP36L1 Enhances Microglial Ferroptosis in Ischemic Stroke by Reducing FTO‐Mediated N6‐Methyladenosine Demethylation of ACSL1 mRNA

**DOI:** 10.1002/kjm2.70212

**Published:** 2026-04-29

**Authors:** Ai‐Xia Song, Han‐Xu Jin, Yuan‐Xin Sun, Wen‐Hui Kou, Xiao‐Ning Niu, Huan‐Huan Wang, Ting‐Ting Zhong, Ping Gu

**Affiliations:** ^1^ Department of Neurology The First Hospital of Hebei Medical University Shijiazhuang Hebei Province People's Republic of China; ^2^ Department of Neurology The First Affiliated Hospital of Hebei North University Zhangjiakou Hebei Province People's Republic of China

**Keywords:** ACSL1, ferroptosis, FTO, ischemic stroke, ZFP36L1

## Abstract

Microglia play an important role in ischemic stroke (IS). However, the molecular regulatory mechanisms underlying microglial ferroptosis in IS remain incompletely understood. In this study, blood samples were collected from 20 IS patients and 15 healthy volunteers. Microglia BV‐2 subjected to oxygen–glucose deprivation/reoxygenation (OGD/R) and mice undergoing middle cerebral artery occlusion‐reperfusion (MCAO/R) surgery were used to establish IS models. Our results revealed that in blood samples from IS patients and in OGD/R‐treated BV‐2 cells, ZFP36 ring finger protein like 1 (ZFP36L1) and Acyl‐CoA synthetase long chain family member 1 (ACSL1) expression was increased, while the fat mass and obesity‐associated (FTO) protein expression was decreased. ACSL1 silencing attenuated ferroptosis and inflammation in OGD/R‐treated microglia, as evidenced by decreased levels of malondialdehyde, Fe^2+^, lipid peroxidation, inflammatory factors, along with an increased Glutathione/Glutathione disulfide ratio. Additionally, ZFP36L1 silencing suppressed the OGD/R‐induced promotion of ferroptosis and inflammation in microglia and alleviated cerebral ischemic injury in MCAO/R mice, whereas ACSL1 overexpression reversed these alterations caused by ZFP36L1 silencing. Mechanistically, ZFP36L1 decreased FTO messenger RNA (mRNA) stability and reduced FTO expression. FTO overexpression reduced ACSL1 N6‐methyladenosine (m6A) modification and ACSL1 expression. In conclusion, ZFP36L1 increased ACSL1 m6A modification and ACSL1 expression to promote microglial ferroptosis, neuroinflammatory response, and cerebral ischemic injury in IS by reducing FTO mRNA stability.

**Abbreviations**
ACSL1: Acyl coenzyme A synthetase long chain family member 1
ACSLs: Acyl‐coenzyme A synthases
BCA: bicinchoninic acid
CCK‐8: Cell Counting Kit‐8
DMEM: Dulbecco's Modified Eagle Medium
ELISA: Enzyme‐linked immunosorbent assay
FTO: Fat mass and obesity‐associated protein
GO: gene ontology
GSEA: Gene set enrichment analysis
GSH: Glutathione
GSSG: Glutathione disulfide
IL‐1*β*
: interleukin‐1β
IL‐6: interleukin‐6
IS: Ischemic stroke
m6A: N6‐methyladenosine
MCAO/R: Middle cerebral artery occlusion‐reperfusion
MDA: Malondialdehyde
MeRIP: Methylated RNA immunoprecipitation
METTL3: m6A methyltransferase‐like 3
mRNA: messenger RNA
NC: negative control
oe: overexpression
OGD/R: Oxygen–glucose deprivation/reoxygenation
PVDF: polyvinylidene difluoride
RIP: RNA immunoprecipitation
RT‐qPCR: Quantitative real‐time polymerase chain reaction
sh‐RNA: Short hairpin RNAs
TNF‐*α*
: tumor necrosis factor‐α
TTC: 2,3,5‐Triphenyltetrazolium Chloride
WTAP: Wilms tumor 1‐associated protein
ZFP36L1: ZFP36 ring finger protein like 1

## 1 Introduction

Ischemic stroke (IS) is a cerebrovascular disorder with complex pathological processes. It can be caused by various factors, and sudden interruption of cerebral blood flow leads to brain metabolic disorders, ischemia, hypoxia, neuronal damage, and functional abnormalities [1]. Thrombolytic and interventional therapies are currently the most effective interventions for patients with IS. However, due to the narrow time window of thrombolytic drugs, as well as the risks and complications of surgery, a large number of patients with IS cannot receive timely and effective treatment, and the probability of vascular recanalization remains low [2]. In addition, reperfusion injury caused by vascular recanalization is a serious problem that must be considered in IS treatment [3]. Therefore, exploring new interventions and targets for IS is urgently needed.

Microglia, the resident immune cells widely distributed in the brain, have a decisive influence on the physiological and pathological environment of the brain, including performing immune surveillance functions, maintaining the integrity of the blood–brain barrier, and responding to brain injury [4]. It has been reported that when IS occurs, microglia sense and respond immediately, participating in many processes such as inflammation, neuronal damage, and injury repair [5]. Microglia exhibit different functional states at different stages after IS, allowing them to play dual roles in neuronal damage and neuroprotection [6]. As previously described, activated microglia release inflammatory factors and thereby induce neuronal tissue damage, while also secreting anti‐inflammatory and neurotrophic factors to promote the recovery of nerve function [5]. Therefore, regulating microglial function and inhibiting inflammatory responses are critical to alleviating IS. Ferroptosis is a recently identified form of programmed cell death that is dependent on iron, accompanied by a large accumulation of membrane lipid peroxides and elevated levels of reactive oxygen species [7]. Previous evidence has linked ferroptosis to a variety of neurological conditions, such as neurodegenerative diseases, hemorrhagic stroke, and IS [8]. Notably, the accumulation of iron in microglia promotes reactive oxygen species release, thereby aggravating neurological injury after IS [9]. Therefore, it is essential to investigate ferroptosis in microglia and its underlying molecular regulatory mechanisms.

Acyl coenzyme A synthetase long‐chain family member 1 (ACSL1), a key lipid metabolic enzyme, is involved in fatty acid metabolism in many tissues, including adipose tissue, heart, liver, and other organs [10]. As previously documented, ACSL1 is involved in the regulation of inflammation [11]. Some evidence has revealed that ACSL1 contributes to ferroptosis by increasing lipid peroxidation [12]. In Severe Acute Respiratory Syndrome Coronavirus 2 infection‐induced IS, ACSL1 was reported to be upregulated, a finding validated through single‐cell RNA sequencing and bioinformatics analysis [13]. Analysis of the GSE16561 dataset revealed that ACSL1 expression was abnormally high in the peripheral blood of patients with IS. This evidence indicates that ACSL1 accelerates IS progression by promoting ferroptosis and inflammation in microglia. Notably, N6‐methyladenosine (m6A), a common RNA methylation modification, occurs in almost all types of RNA [14]. A previous review highlighted the important role of m6A modifications in IS [15]. More importantly, multiple m6A modification sites on ACSL1 messenger RNA (mRNA) have been predicted, suggesting that the abnormally high expression of ACSL1 in IS may be regulated by m6A modification and the key enzymes mediating this process.

ZFP36 ring finger protein‐like 1 (ZFP36L1), a member of the ZFP36 family, is an RNA‐binding protein. Two zinc finger structures in ZFP36L1 bind homologous RNA domains, which specifically recognize and bind the adenylate‐uridylate‐rich elements of the mRNA 3′ untranslated region and exert various post‐transcriptional regulatory functions, including mRNA degradation [16]. ZFP36L1 has been implicated in inflammation [17]. Through gene set enrichment analysis (GSEA), we found that ZFP36L1 was one of the genes associated with ferroptosis. Moreover, the GSE16561 dataset showed an abnormally high expression of ZFP36L1 under IS conditions, which was positively correlated with ACSL1. However, few studies have investigated ZFP36L1 in IS, and it is unclear whether ZFP36L1 affects the IS by regulating ferroptosis and inflammation. In addition, through bioinformatics analysis, we found that ZFP36L1 can bind to a variety of m6A‐modifying enzymes, such as m6A methyltransferase‐like 3 (METTL3), fat mass and obesity‐associated protein (FTO), Wilms tumor 1‐associated protein (WTAP), and AlkB homolog 5. Thus, we speculated that ZFP36L1 might be involved in IS progression by targeting the mRNA of specific m6A‐modifying enzymes for degradation.

In this study, the influence and molecular mechanism of ACSL1 in regulating microglial inflammatory response and ferroptosis were investigated. We hypothesized that ACSL1 is regulated by ZFP36L1‐mediated stability of m6A modifying enzymes, thereby participating in IS progression. We combined bioinformatic data, clinical sample collection, cell experiments, and animal experiments to verify our hypothesis. The experimental results of this study will help clarify the pathological molecular regulatory mechanisms of IS.

## 2 Methods

### 2.1 Peripheral Blood Collection in IS Patients

In this study, 20 patients with IS and 15 healthy volunteers were recruited from the First Affiliated Hospital of Hebei North University, and their peripheral blood was collected. Patients with IS were diagnosed using magnetic resonance imaging combined with clinical signs and symptoms, such as focal or global loss of brain function and new cerebral infarction. Patients with coronary artery disease, kidney disease, autoimmune disease, and circulatory disorders were excluded. This study was approved by the Medical Ethics Committee of First Affiliated Hospital of Hebei North University (K202418P). All participants provided written informed consent.

### 2.2 Cell Culture

BV‐2 cells, a cell line of mouse microglia (Shanghai Cell Bank, China), were cultured in Dulbecco's modified Eagle's medium (DMEM; Gibco, USA) supplemented with 10% fetal bovine serum (Gibco) and 1% antibiotics (Beyotime, China). Cells were grown in an incubator at 5% CO_2_ & 37°C.

Oxygen–glucose deprivation/reoxygenation (OGD/R) was applied to mimic IS in vitro. First, glucose‐free DMEM cultured BV‐2 cells were placed in an incubator with 5% CO_2_ and 95% N_2_ for 2 h. After 2 h, BV‐2 cells were returned to normal DMEM and cultured under normal conditions for 48 h.

### 2.3 Cell Transfection

Short hairpin RNAs (sh‐RNAs) targeting ACSL1 (sh‐ACSL1#1, sh‐ACSL1#2), ZFP36L1 (sh‐ZFP36L1), overexpression (oe) plasmids including oe‐ACSL1 and oe‐FTO and controls including sh‐RNA negative control (sh‐RNA) and oe‐negative control (oe‐NC) were purchased from GenePharma (Shanghai, China). According to our experimental design, BV‐2 cells were transfected with the above plasmids for 48 h using Lipofectamine 3000 (Invitrogen, USA).

### 2.4 Reverse Transcription Quantitative Real‐Time Polymerase Chain Reaction (RT‐qPCR)

Total RNAs were extracted from the peripheral blood of patients with IS, BV‐2 cells, and mouse brain tissue using TRIzol (Takara, Japan). An RNA reverse transcription kit (Takara) was used to reverse transcribe total RNA into complementary DNA. SYBR Premix Ex Taq (Takara) was used to perform qPCR using an ABI Prism 7500 RT‐PCR system (Applied Biosystems, USA). Relative gene expression was normalized to *β*‐actin using the 2^−ΔΔCt^ method. The primers are shown in (Table 1).

**TABLE 1:** Primer sequences used for RT‐qPCR.

Gene name	Forward	Reverse
ACSL1 (human)	CCATGAGCTGTTCCGGTATTT	CCGAAGCCCATAAGCGTGTT
ZFP36L1 (human)	ACTCCAGCCGCTACAAGAC	CGTAGGGGCAAAAGCCGAT
TNF‐*α* (human)	CCCCAGGGACCTCTCTCTAA	TGAGGTACAGGCCCTCTGAT
IL‐1*β* (human)	CGATGCACCTGTACGATCAC	TCTTTCAACACGCAGGACAG
IL‐6 (human)	ACAGGGAGAGGGAGCGATAA	GAGAAGGCAACTGGACCGAA
*β*‐Actin (human)	TGGCACCACACCTTCTACAA	CCAGAGGCGTACAGGGATAG
TNF‐*α* (mouse)	CCGATGGGTTGTACCTTGTC	TGGAAGACTCCTCCCAGGTA
IL‐1*β* (mouse)	GCCACCTTTTGACAGTGATGAG	AAGGTCCACGGGAAAGACAC
IL‐6 (mouse)	TGCAAGAGACTTCCATCCAG	TCCACGATTTCCCAGAGAAC
METTL3 (mouse)	TGACTCCAGTGCTGATCGAC	CTGCGCATCTCATCATCTGT
METTL14 (mouse)	GAGCTGAGAGTGCGGATAGC	GCAGATGTATCATAGGAAGCCC
FTO (mouse)	GACACTTGGCTTCCTTACCTG	CTCACCACGTCCCGAAACAA
ACSL1 (mouse)	TGCCAGAGCTGATTGACATTC	GGCATACCAGAAGGTGGTGAG
ZFP36L1 (mouse)	GTGTCCGCCACCATTTTTGAC	CGCTGGGAGTGCTGTAGTTG
*β*‐Actin (mouse)	GGCTGTATTCCCCTCCATCG	CCAGTTGGTAACAATGCCATGT

### 2.5 Cell Counting Kit‐8 (CCK‐8) Assay

BV‐2 cells were seeded into 96‐well plates at a density of 1 × 10^4^ cells/well. After 24 h, 10 μL of CCK‐8 solution was added to each well and incubated for 2 h at 37°C. The absorbance was then measured at 450 nm using a microplate reader.

### 2.6 Enzyme‐Linked Immunosorbent Assay (ELISA)

According to the manufacturer's instructions, the levels of tumor necrosis factor‐*α* (TNF‐*α*), interleukin‐1*β* (IL‐1*β*), and interleukin‐6 (IL‐6) in the peripheral blood of patients with IS and in the BV‐2 cell culture supernatants were measured using ELISA kits (PT518/PT512, PI305/PI301, PI325/PI326; Beyotime).

### 2.7 Detection of Malondialdehyde (MDA) and Glutathione (GSH)/glutathione Disulfide (GSSG) Ratio

Brain tissues from mice were homogenized, and the peripheral blood of patients with IS and BV‐2 cells was collected. After centrifugation, the supernatants were acquired and the MDA level and the ratio of GSH/GSSG were measured following the manufacturer's instructions. The MDA (A003‐1‐2) and GSH/GSSG assay kits were purchased from Nanjing Jiancheng (China).

### 2.8 Detection of Fe^2+^ Levels

A FerroOrange probe (F374, Dojindo, China) was used to detect the Fe^2+^ levels in BV‐2 cells. Briefly, BV‐2 cells were seeded into 96‐well plates (1 × 10^4^ cells/well) and incubated at 37°C and 5% CO_2_ for 24 h. Subsequently, the cells were incubated at 37°C in the dark with 1 μM FerroOrange probe for 30 min after washing with buffer. A microplate reader (Bio‐Rad, USA) was used to detect the fluorescence intensity at 543 nm (excitation) and 580 nm (emission).

### 2.9 Western Blot

BV‐2 cells with different transfections and brain tissues from mice were lysed using radioimmunoprecipitation assay buffer (Beyotime). After determining the concentrations of the extracted proteins using a bicinchoninic acid (BCA) assay, equal quantities of protein were loaded into the gel wells, and proteins were separated by electrophoresis. Separated proteins were transferred onto polyvinylidene difluoride (PVDF) membranes. After blocking with 5% skimmed milk for 1 h, PVDF membranes were incubated with primary antibodies of ACSL1 (PA5‐17136, 1:1000, Invitrogen), FTO (PA5‐89791, 1:1000, Invitrogen), ZFP36L1 (ZFP36L1‐101AP, 1:1000, Fabgennix, USA), and *β*‐actin (PA1‐183, 1:5000, Invitrogen) overnight at 4°C. The next day, secondary antibodies (31,460, 1:50000, Invitrogen) were used to incubate the PVDF membranes for 2 h after washing. Finally, to visualize protein bands, enhanced chemiluminescence reagent (Thermo Fisher Scientific, USA) was used. The band intensities were analyzed using Image J software.

### 2.10 Detection of Lipid Peroxidation

BODIPY 581/591 C11 (D3861, Thermo Fisher Scientific) was used to detect lipid peroxidation in BV‐2 cells. Briefly, BV‐2 cells were seeded in 6‐well plates (2 × 10^5^ cells/well) and cultured at 37°C and 5% CO_2_ for 24 h. Subsequently, C11‐BODIPY (10 μM) solution was added and incubated in the dark at 37°C for 30 min after washing. Then, BV‐2 cells were digested with 0.25% trypsin, washed, and resuspended in PBS solution. Finally, lipid peroxidation was measured using flow cytometry.

### 2.11 Methylated RNA Immunoprecipitation (MeRIP)

The Magna MeRIP m6A Kit (17–10,499, Millipore, Germany) was used to conduct MeRIP experiments, following the manufacturer's protocol. BV‐2 cells treated according to the experimental design were lysed using RNA immunoprecipitation (RIP) lysis buffer, and total RNA was extracted. Subsequently, immunoprecipitates in the RNA samples were obtained using magnetic beads coated with anti‐m6A (MA5‐33030, Invitrogen) or anti‐immunoglobulin G (ab172730, Abcam). The co‐precipitated RNAs were then purified and dissolved in RNase‐free water. The m6A enrichment of ACSL1 mRNA was detected using RT‐qPCR.

### 2.12 RIP

An RIP assay was performed, and a Magna RIP RNA‐Binding Protein Immunoprecipitation Kit (Millipore, USA) was used to validate the interaction between FTO and ACSL1 mRNA, and the interaction between ZFP36L1 and FTO mRNA in BV‐2 cells. Briefly, BV‐2 cells were lysed in the RIP Lysis Buffer. After centrifugation, anti‐FTO (PA5‐89791, Invitrogen), anti‐ZFP36L1 (ZFP36L1‐101AP, Fabgennix), and anti‐immunoglobulin G (ab172730, Abcam) were used to incubate lysates overnight at 4°C. The prepared Protein A/G magnetic beads (Thermo Fisher Scientific) were added to the samples and incubated with antibodies for 1 h. The samples were then subjected to centrifugation. The precipitates were suspended in protease K buffer, and TRIzol was used to extract RNA. The levels of RNAs precipitated by antibodies against FTO and ACSL1 were measured using RT‐qPCR.

### 2.13 mRNA Stability of ACSL1 and FTO

BV‐2 cells were subjected to various treatments. Actinomycin D (ActD, 5 μg/mL), an inhibitor of mRNA transcription, was applied to incubate BV‐2 cells for 0, 2, 4, 6, and 8 h. Finally, the mRNA levels of ACSL1 and FTO in BV‐2 cells were extracted at different time points after ActD treatment and analyzed by RT‐qPCR.

### 2.14 RNA Pull‐Down Assay

Biotin‐labeled FTO (Bio‐FTO) was obtained from Sangon Biotech (China). A Pierce Magnetic RNA‐Protein Pull‐Down Kit (Thermo Fisher Scientific) was used for RNA pull‐down. After probe hybridization, BV‐2 cells were incubated with streptavidin‐coated magnetic beads. Subsequently, proteins were pulled down using beads and detected by Western blot.

### 2.15 Middle Cerebral Artery Occlusion‐Reperfusion (MCAO/R) Model Construction

Male C57BL/6 mice (8–10 weeks old; 22–24 g body weight) were bought from Hunan Slac Jingda Laboratory Animal Co. Ltd. and housed in a standard temperature and humidity environment. All mice were provided with food and water ad libitum. After 1 week of adaptive feeding, mice were randomly divided into five groups: Sham, MCAO/R, MCAO/*R* + sh‐NC + oe‐NC, MCAO/*R* + sh‐ZFP36L1 + oe‐NC, and MCAO/*R* + sh‐ZFP36L1 + oe‐ACSL1. Five mice were assigned to each group. The mice were anesthetized by intraperitoneal injection of pentobarbital sodium (50 mg/kg). Except for the mice in the sham group, mice in the other groups underwent MCAO surgery, and blood flow was allowed to recover 2 h after the operation. According to a previous study [18], focal cerebral ischemia was induced by occlusion of the right middle cerebral artery with a 6–0 nylon monofilament. After 2 h of occlusion, the nylon monofilaments were removed to restore blood flow. Mice in the Sham group underwent the same procedure as those in the MCAO/R group, but their blood flow remained unoccluded. Three days before the MCAO/R operation, adenoviruses containing sh‐ZFP36L1, oe‐ACSL1, or their negative control plasmid (2 × 10^8^ PFU/μL, 5 μL), purchased from GenePharma, were injected into mice brain via left lateral ventricle injection. Following anesthesia, the mice were fixed in a stereotaxic frame. The adenovirus solution was administered to the mice using a microsyringe. Injections targeted three specific brain coordinates relative to Bregma: anteroposterior +1.0 mm, mediolateral −0.59 mm, and dorsoventral −2 mm. The surgical procedure was completed by suturing the skin incision. The neurological deficit score of the mice was calculated 24 h after the MCAO/R operation. Then, after euthanizing, the brain tissues were collected for subsequent experiments. Animal experiments were approved by the Committee of Experimental Animals of Hebei North University (HBNU20240822122).

### 2.16 2,3,5‐Triphenyltetrazolium Chloride (TTC) Staining

Cerebral infarction in mice was evaluated using TTC staining. The specific steps were as follows: Mouse brain tissue was cut into slices of approximately 2 mm. The slices were then stained with 2% TTC solution (Millipore) at 37°C for 10 min. After fixation with 4% paraformaldehyde for 24 h, Image J software (National Institutes of Health, USA) was used to measure the infarcted areas, and the infarct volume percentage was further determined by dividing the infarct volume by the total contralateral hemispheric volume and multiplying by 100%.

### 2.17 Neurological Deficit Score

Neurological deficit scores were evaluated in all mice at 24 h after MCAO/R by an investigator who was blinded to the experimental groups. The neurological deficit score was measured via a five‐point scale [19]: a score of zero indicated no neurological deficit; a score of one (failure to fully extend the left forepaw) indicated a mild focal neurological deficit, a score of two (circling to the left) indicated a moderate focal neurological deficit, a score of three (falling to the left) indicated a severe focal deficit, and a score of four indicated an inability to walk spontaneously and a depressed level of consciousness. The higher the neurological deficit score, the more severe the neurological impairment.

### 2.18 Nissl Staining

Dehydrated mouse brain tissue was embedded in paraffin wax. The paraffin blocks were then cut into slices of about 6 μm. Subsequently, the sections were incubated with Nissl stain solution (Solarbio, China) for 10 min at room temperature. After dehydration and mounting with neutral balsam, the stained sections were examined under an Olympus microscope to observe the morphology of mouse brain tissue after IS.

### 2.19 Data Collection and Gene Ontology (GO) Analysis

The GSE16561 dataset was obtained from the GEO database (https://www.ncbi.nlm.nih.gov/geo/). The DAVID 6.8 program (https://david.ncifcrf.gov/) was used to analyze the GO functional annotations. The SRAMP database (http://www.cuilab.cn/sramp) was used to predict m6A modification sites on ACSL1 mRNA.

### 2.20 Data Analysis

All data were presented as means ± standard deviation. All data were analyzed using GraphPad Prism version 9. An a priori power analysis was conducted using G*Power to determine the sample size required to achieve 80% statistical power for detecting a significant treatment effect at *α* = 0.05. Comparisons between two groups were conducted using Student's *t*‐test, while comparisons among three or more groups were performed using one‐way analysis of variance followed by Tukey's post hoc test. Relationships between gene expression levels were established using Pearson's correlation analysis. *p* < 0.05 was regarded as statistically significant.

## 3 Results

### 3.1 ACSL1 Was Highly Expressed in Blood Samples of IS Patients and OGD/ R‐Induced Microglia

By analyzing the GSE16561 dataset, differentially expressed genes in the peripheral blood of 24 healthy individuals and 39 IS patients were identified Figure S1A. The differentially expressed genes associated with IS were closely related to the immune response, as analyzed by GO functional enrichment Figure S1B. Subsequently, peripheral blood samples were collected from 15 healthy volunteers and 20 patients with IS. Measurement of peripheral blood revealed that levels of pro‐inflammatory factors, including TNF‐*α*, IL‐1*β*, and IL‐6, were significantly higher in IS samples than in those from healthy volunteers Figure S1C. Given the close association between IS and inflammatory responses, we subsequently focused on microglia, the key regulators of neuroinflammation. As shown in Figure S1D, OGD/R treatment decreased the viability of BV‐2 cells. Levels of pro‐inflammatory factors (TNF‐*α*, IL‐1*β*, and IL‐6) in the supernatants of OGD/R‐induced microglia were markedly elevated. Figure S1E,F showed 10 genes that were abnormally highly expressed in the peripheral blood of IS patients in the GSE16561 database, and ACSL1 was one of the 10 genes. What's more, GSEA plot showed that ACSL1 expression is positively correlated with ferroptosis in the GSE16561 database Figure S1G. To further explore whether the abnormally high expression of ACSL1 was associated with IS, we collected blood samples from patients with IS. The results showed that ACSL1 expression in the peripheral blood of IS patients was higher than that in healthy volunteers Figure S1H. ACSL1 is a ferroptosis‐related gene [20]; therefore, we examined whether its abnormal expression is associated with ferroptosis in microglia. Further experimental results indicated that MDA levels were increased, and GSH/GSSG ratio was downregulated in the peripheral blood of patients with IS Figure S1I. As expected, enhanced ACSL1 expression was observed in BV‐2 cells subjected to OGD/R Figure S1J,K. OGD/R exposure also resulted in elevated MDA and Fe^2+^ levels and decreased GSH/GSSG ratio Figure S1L–N. Collectively, ACSL1 likely plays a key role in IS, potentially through microglial ferroptosis.

### 3.2 ACSL1 Knockdown Suppressed OGD/R‐Induced Ferroptosis and Inflammation in Microglia

Then we investigated the effect of ACSL1 on ferroptosis and inflammation in OGD/R‐induced microglia. First, sh‐ACSL1#1 and sh‐ACSL1#2 were used to transfect BV‐2 cells, resulting in a significant decrease in ACSL1 expression (Figure 1A,B). Figure 1C,D showed that ACSL1 knockdown reversed the OGD/R‐induced increase in ACSL1 expression in microglia. In addition, OGD/R‐induced impairment of cell viability was alleviated by ACSL1 knockdown (Figure 1E). The elevated levels of TNF‐*α*, IL‐1*β*, and IL‐6 caused by OGD/R exposure were abolished by ACSL1 knockdown (Figure 1F,G). Moreover, OGD/R exposure increased MDA and Fe^2+^ levels and decreased the GSH/GSSG ratio in BV‐2 cells; these effects were offset by ACSL1 knockdown (Figure 1H–J). We also observed that OGD/R elevated lipid peroxidation levels, which were eliminated by ACSL1 knockdown in BV‐2 cells (Figure 1K). Taken together, ACSL1 knockdown inhibited OGD/R‐induced ferroptosis and inflammation in microglia.

**FIGURE 1:**
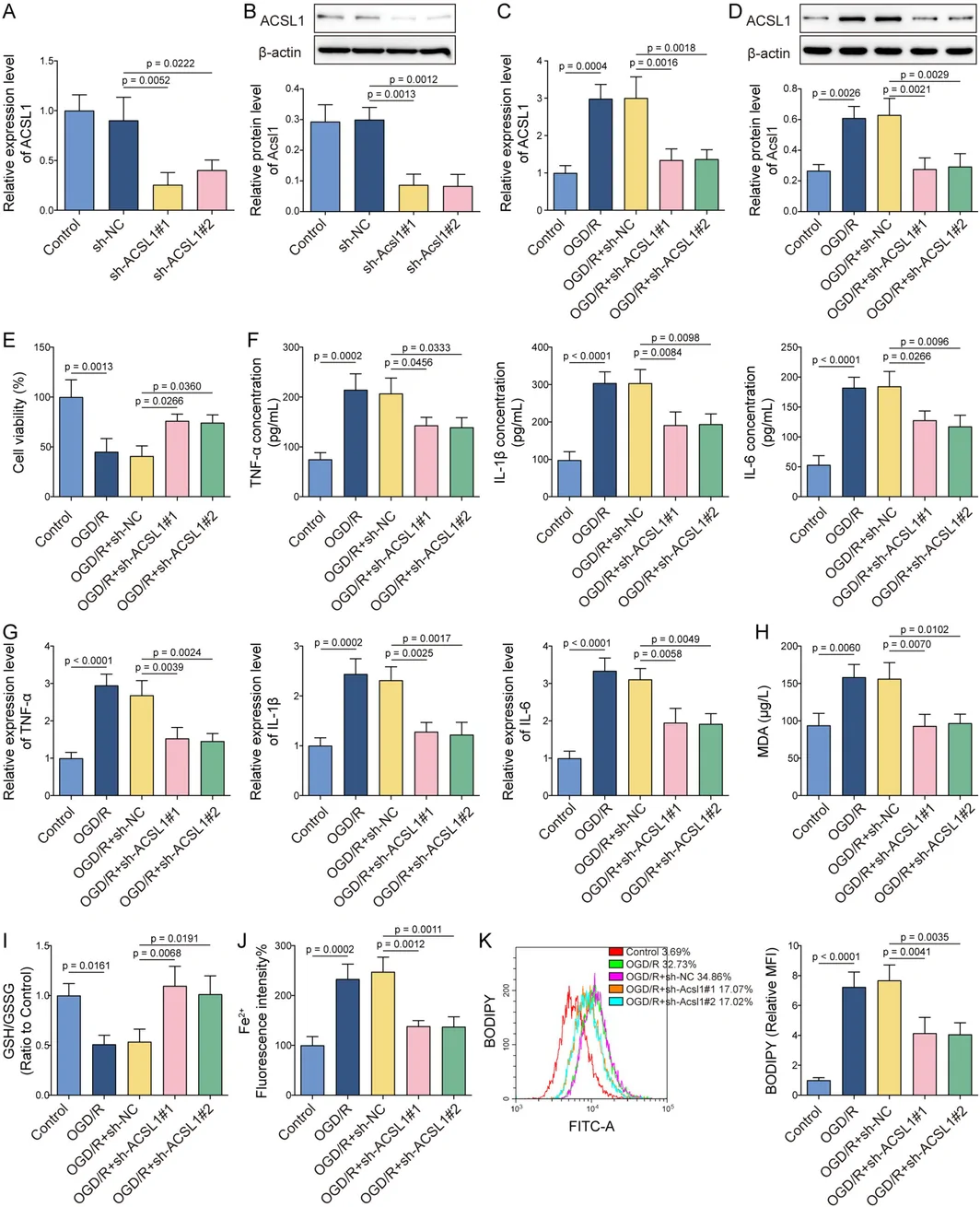
ACSL1 knockdown suppressed OGD/R‐induced ferroptosis and inflammation in microglia. BV‐2 cells were transfected with either sh‐ACSL1#1 or sh‐ACSL1#2. (A‐B) ACSL1 expression was measured using RT‐qPCR and western blot. BV‐2 cells transfected with sh‐ACSL1#1 and sh‐ACSL1#2 were subjected to OGD/R. (C‐D) ACSL1 expression was measured using RT‐qPCR and western blot, (E) Cell viability was evaluated using the CCK‐8 assay. (F‐G) TNF‐*α*, IL‐1*β*, and IL‐6 levels in the supernatants were determined using ELISA and RT‐qPCR. (H‐I) MDA levels and GSH/GSSG ratios were examined using the corresponding assay kits. (J) Fe^2+^ levels were measured using the FerroOrange probe. (K) Lipid peroxidation levels were measured using the C11‐BODIPY 581/591 fluorescent probe. All experiments were conducted 3 times independently, *n* = 3 per group.

### 3.3 FTO Weakened the Stability of ACSL1 mRNA by Reducing m6A Modification of ACSL1

Multiple m6A modification sites on ACSL1 mRNA were predicted (Figure 2A). Thus, the levels of m6A methyltransferases (METTL3 and METTL14) and the demethylase FTO were measured in the collected clinical samples. The results showed that METTL3 and FTO expression were lower, whereas METTL14 expression was higher in the IS samples (Figure 2B). Pearson correlation analysis was performed to evaluate the correlations of METTL3, METTL14, and FTO expression with ACSL1 expression in IS samples. Only a negative correlation between FTO and ACSL1 expression was observed, whereas no correlation was found between the others and ACSL1 expression (Figure 2C). Additionally, OGD/R increased m6A‐modified ACSL1 levels in BV‐2 cells, as determined using MeRIP (Figure 2D). As expected, OGD/R decreased FTO expression in BV‐2 cells (Figure 2E,F). RIP assay validated the interaction between FTO and ACSL1 mRNA in BV‐2 cells, in which the FTO antibody enriched ACSL1 mRNA (Figure 2G). In addition, FTO expression was significantly increased in BV‐2 cells after oe‐FTO transfection (Figure 2H,I). MeRIP results showed that FTO overexpression markedly reduced the m6A‐modified ACSL1 level (Figure 2J). After ActD treatment, FTO overexpression decreased the stability of ACSL1 mRNA (Figure 2K). Next, oe‐FTO‐transfected BV‐2 cells were treated with OGD/R. As shown in (Figure 2L,M), OGD/R induction decreased FTO expression and increased ACSL1 expression, whereas FTO overexpression partially reversed these changes. Moreover, OGD/R upregulated m6A modification of ACSL1 mRNA, which was reversed by FTO overexpression (Figure 2N). As shown in (Figure 2O), OGD/R stabilized ACSL1 mRNA, and FTO overexpression abolished this effect. Overall, FTO decreased m6A‐modified ACSL1 levels and reduced ACSL1 mRNA stability in OGD/R‐treated BV‐2 cells.

**FIGURE 2:**
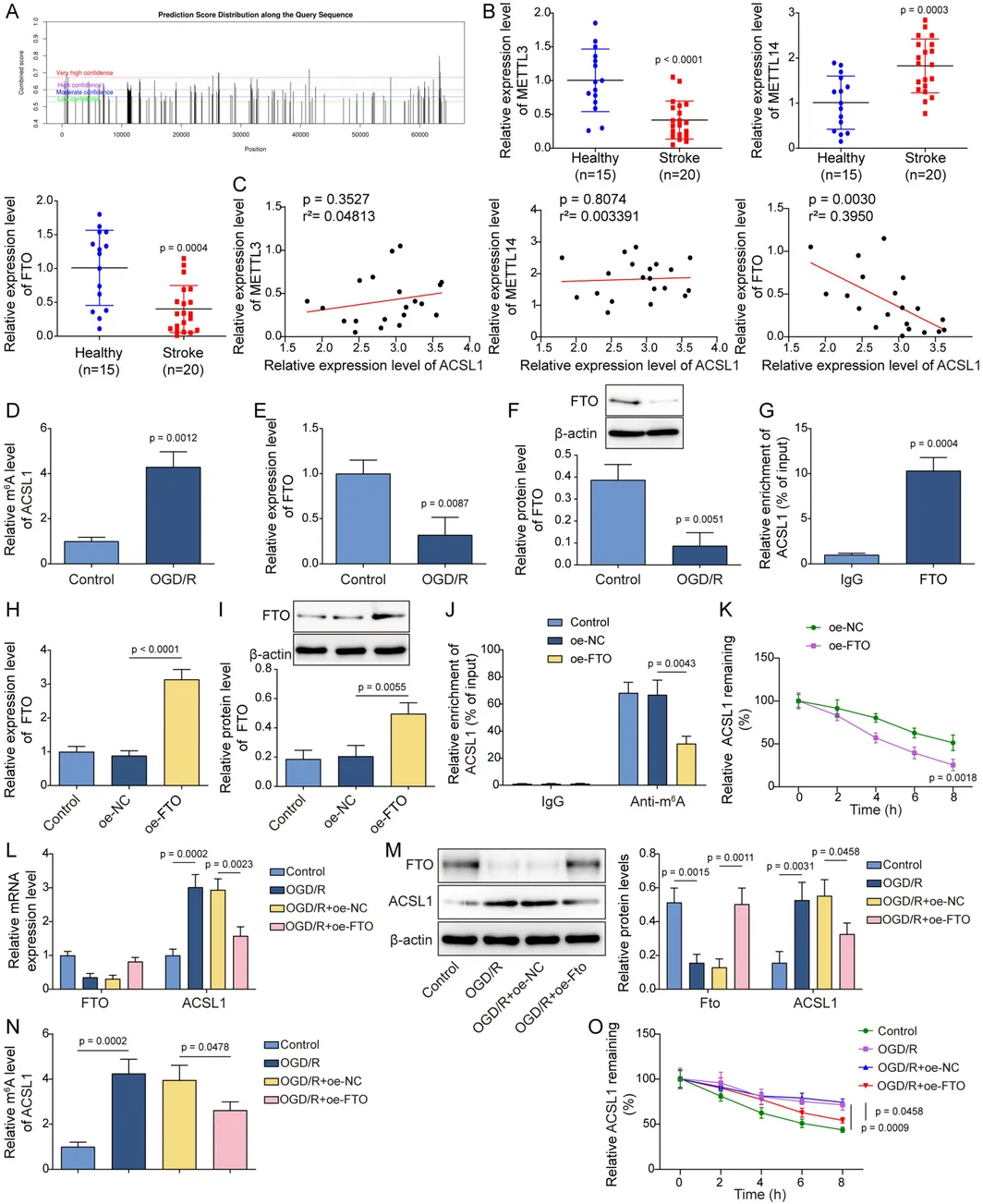
FTO weakened the stability of ACSL1 mRNA by reducing the m6A‐modified ACSL1. (A) m6A modification sites of ACSL1 were predicted using the SRAMP database. (B) The levels of METTL3, METTL14, and FTO were detected in clinical samples using RT‐qPCR (Healthy: *N* = 15, Stroke: *N* = 20). (C) The relationships of METTL3, METTL14, and FTO expression with ACSL1 expression in IS samples were analyzed by Pearson correlation analysis (*n* = 20). (D) The level of m6A‐modified ACSL1 in OGD/R‐treated BV‐2 cells was examined using MeRIP. (E‐F) FTO expression was measured in OGD/R‐treated BV‐2 cells using RT‐qPCR and western blot. (G) The interaction between FTO and ACSL1 mRNA was validated using the RIP assay. BV‐2 cells were transfected with oe‐FTO. (H‐I) FTO expression was measured using RT‐qPCR and western blot. (J) The m6A‐modified ACSL1 level in BV‐2 cells with oe‐FTO transfection was examined using MeRIP. (K) The stability of ACSL1 mRNA in BV‐2 cells with oe‐FTO transfection after ActD treatment was measured using RT‐qPCR. Oe‐FTO‐transfected BV‐2 cells were subjected to OGD/R exposure. (L‐M) FTO and ACSL1 expression were evaluated using RT‐qPCR and western blot. (N) The m6A modification level of ACSL1 mRNA was measured using MeRIP. (O) The stability of ACSL1 mRNA in BV‐2 cells with indicated transfection after ActD treatment was measured using RT‐qPCR. For D‐O, experiments were conducted 3 times independently, *n* = 3 per group.

### 3.4 ZFP36L1 Accelerated FTO Degradation and Enhanced m6A‐Modified ACSL1 Levels and ACSL1 Expression by Binding to FTO mRNA

As shown in (Figure 3A), ZFP36L1 levels were higher in the peripheral blood of IS patients than in healthy volunteers. A positive correlation was observed between ZFP36L1 and ACSL1 expression in IS patients (Figure 3B). In addition, ZFP36L1 expression was significantly increased in OGD/R‐treated BV‐2 cells (Figure 3C,D). Figure 3E,F validated the interaction between ZFP36L1 and FTO mRNA. Specifically, the RIP assay showed that the ZFP36L1 antibody enriched the FTO mRNA (Figure 3E). The RNA pull‐down assay demonstrated that the Bio‐FTO probe successfully pulled down ZFP36L1 protein (Figure 3F). BV‐2 cells were transfected with sh‐ZFP36L1, and ZFP36L1 expression was significantly decreased in BV‐2 cells after sh‐ZFP36L1 transfection (Figure 3G,H). However, ZFP36L1 knockdown markedly increased FTO expression (Figure 3I,J) and enhanced FTO mRNA stability (Figure 3K) in BV‐2 cells. Additionally, ZFP36L1 knockdown promoted the interaction between FTO and ACSL1 mRNA, as confirmed by RIP (Figure 3L). As expected, ZFP36L1 knockdown reduced the m6A modification level of ACSL1 mRNA (Figure 3M). Furthermore, sh‐ZFP36L1‐transfected BV‐2 cells were subjected to OGD/R. We observed that OGD/R increased ZFP36L1 and ACSL1 expression and decreased FTO expression in BV‐2 cells, effects reversed by ZFP36L1 knockdown (Figure 3N,O). Taken together, ZFP36L1 increases m6A‐modified ACSL1 levels and ACSL1 expression by reducing FTO mRNA stability and expression.

**FIGURE 3:**
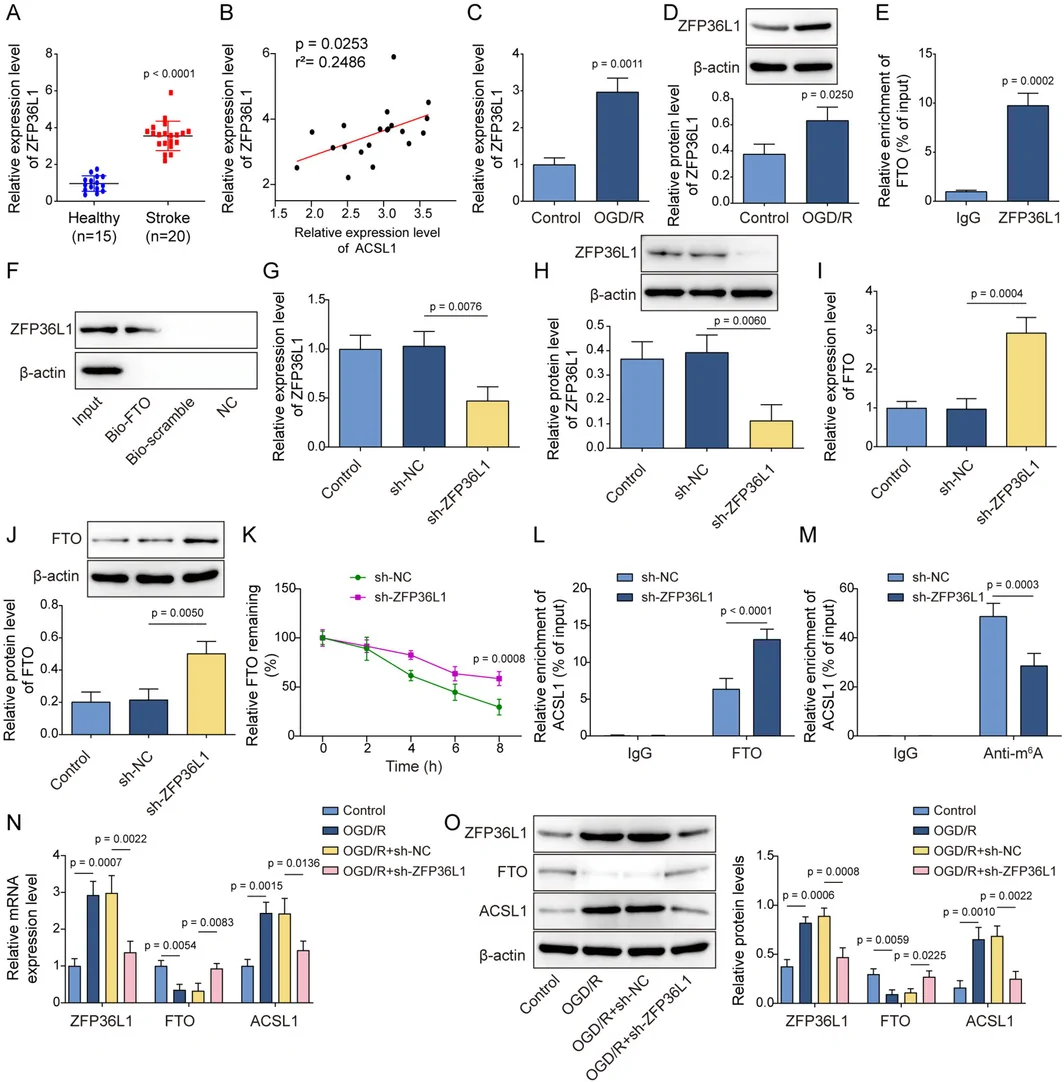
ZFP36L1 accelerated FTO degradation and enhanced m6A‐modified ACSL1 and ACSL1 expression by binding to FTO mRNA. (A) ZFP36L1 expression in clinical samples was assessed using RT‐qPCR (Healthy: *N* = 15, Stroke: *N* = 20). (B) The correlation between ZFP36L1 and ACSL1 expression in IS patients was analyzed using Pearson correlation analysis (*n* = 20). BV‐2 cells were treated with OGD/R exposure. (C‐D) ZFP36L1 expression was assessed using RT‐qPCR and western blot. (E‐F) The interaction between ZFP36L1 and FTO mRNA was verified using RIP and RNA pull‐down. BV‐2 cells were subjected to sh‐ZFP36L1 transfection. (G‐J) ZFP36L1 and FTO expression was examined using RT‐qPCR and western blot. (K) The stability of FTO mRNA in BV‐2 cells with sh‐ZFP36L1 transfection after ActD treatment was measured using RT‐qPCR. (L) The interaction between FTO and ACSL1 mRNA was validated in BV‐2 cells after sh‐ZFP36L1 transfection using the RIP assay. (M) The m6A modification level of ACSL1 mRNA in BV‐2 cells with sh‐ZFP36L1 transfection was examined using MeRIP. sh‐ZFP36L1‐transfected BV‐2 cells were subjected to OGD/R exposure. (N‐O) ZFP36L1, FTO, and ACSL1 expression was detected using RT‐qPCR and western blot. For C‐O, experiments were conducted 3 times independently, *n* = 3 per group.

### 3.5 ZFP36L1 Knockdown Attenuated OGD/R‐Induced Ferroptosis and Inflammation in Microglia

To investigate the influence of ZFP36L1 on ferroptosis and inflammation in microglia, BV‐2 cells were transfected with sh‐ZFP36L1 and subsequently subjected to OGD/R treatment. OGD/R‐impaired cell viability was rescued by ZFP36L1 knockdown (Figure 4A). Additionally, TNF‐*α*, IL‐1*β*, and IL‐6 levels were markedly elevated by OGD/R exposure, whereas ZFP36L1 knockdown attenuated these increases (Figure 4B,C). ZFP36L1 knockdown suppressed the effects of OGD/R on ferroptosis by reducing MDA, Fe^2+^ and lipid peroxidation levels and increasing the GSH/GSSG ratio (Figure 4D–G). Collectively, ZFP36L1 knockdown inhibited OGD/R‐induced ferroptosis and inflammation in microglia.

**FIGURE 4:**
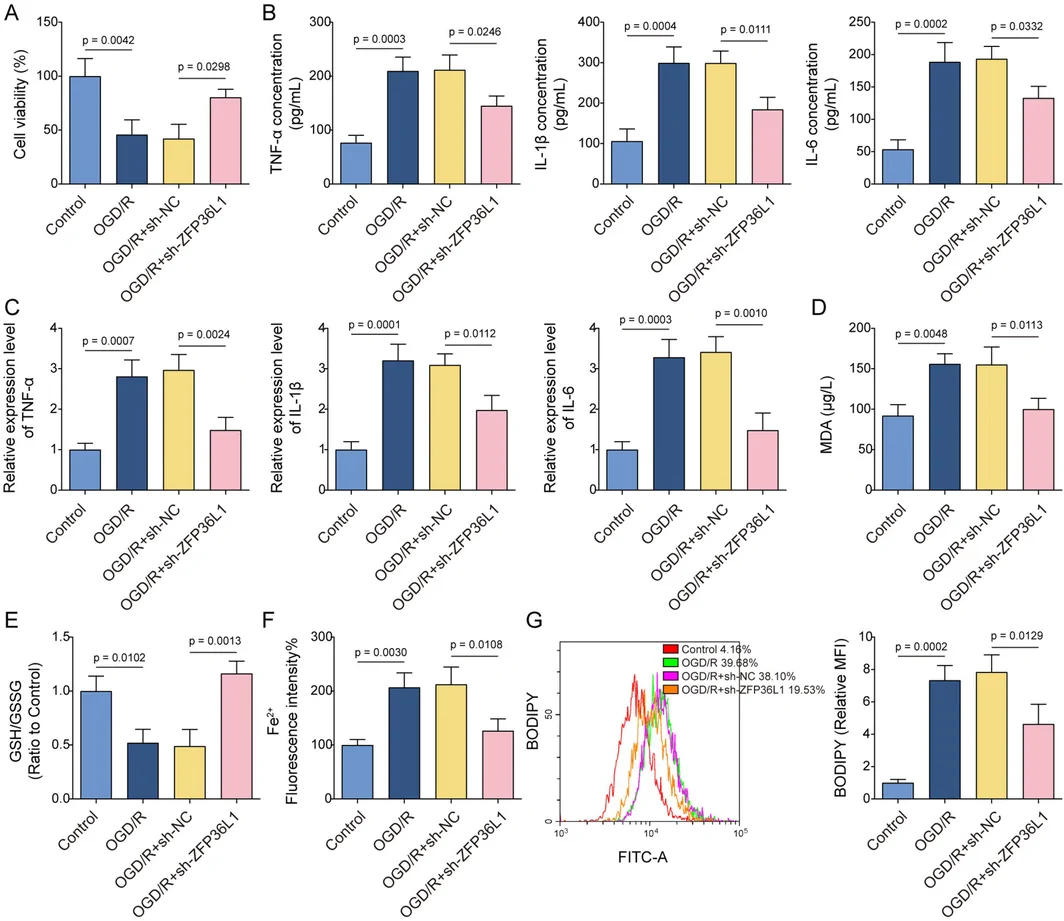
ZFP36L1 knockdown attenuated OGD/R‐induced ferroptosis and inflammation in microglia. BV‐2 cells were transfected with sh‐ZFP36L1 and subjected to OGD/R. (A) Cell viability was evaluated using the CCK‐8 assay. (B‐C) TNF‐*α*, IL‐1*β*, and IL‐6 levels in supernatants were determined using ELISA and RT‐qPCR. (D‐E) MDA levels and GSH/GSSG ratios were examined using the corresponding assay kits. (F) The Fe^2+^ levels were measured using a FerroOrange probe. (G) Lipid peroxidation levels were measured using the C11‐BODIPY 581/591 fluorescent probe. All experiments were conducted 3 times independently, *n* = 3 per group.

### 3.6 ACSL1 Overexpression Reversed ZFP36L1 Knockdown‐Mediated Suppression of Ferroptosis and Inflammation in OGD/R‐Induced Microglia

To investigate whether the ZFP36L1/ACSL1 axis affected ferroptosis and inflammation in OGD/R‐treated microglia, BV‐2 cells were transfected with oe‐ACSL1. (Figure 5A,B) showed the upregulation of ACSL1 expression after oe‐ACSL1 transfection. Subsequently, BV‐2 cells were transfected with sh‐ZFP36L1 or oe‐ACSL1, followed by OGD/R treatment. ZFP36L1 knockdown inhibited ACSL1 expression in OGD/R‐treated microglia, whereas ACSL1 overexpression reversed the ZFP36L1 knockdown‐induced reduction in ACSL1 expression (Figure 5C,D). In addition, ZFP36L1 knockdown increased the viability of BV‐2 cells under OGD/R conditions, which was blocked by ACSL1 overexpression (Figure 5E). Meanwhile, ZFP36L1 knockdown decreased TNF‐*α*, IL‐1*β*, and IL‐6 levels in OGD/R‐induced microglia, and this effect was reversed by ACSL1 overexpression (Figure 5F,G). Furthermore, ACSL1 overexpression effectively reversed the ZFP36L1 knockdown‐mediated reduction in MDA, Fe^2+^ and lipid peroxidation levels and enhanced the GSH/GSSG ratio in BV‐2 cells upon OGD/R treatment (Figure 5H–K). In summary, ZFP36L1 knockdown inhibited ferroptosis and inflammation in OGD/R‐treated microglia by decreasing ACSL1 expression, which could be reversed by ACSL1 overexpression.

**FIGURE 5:**
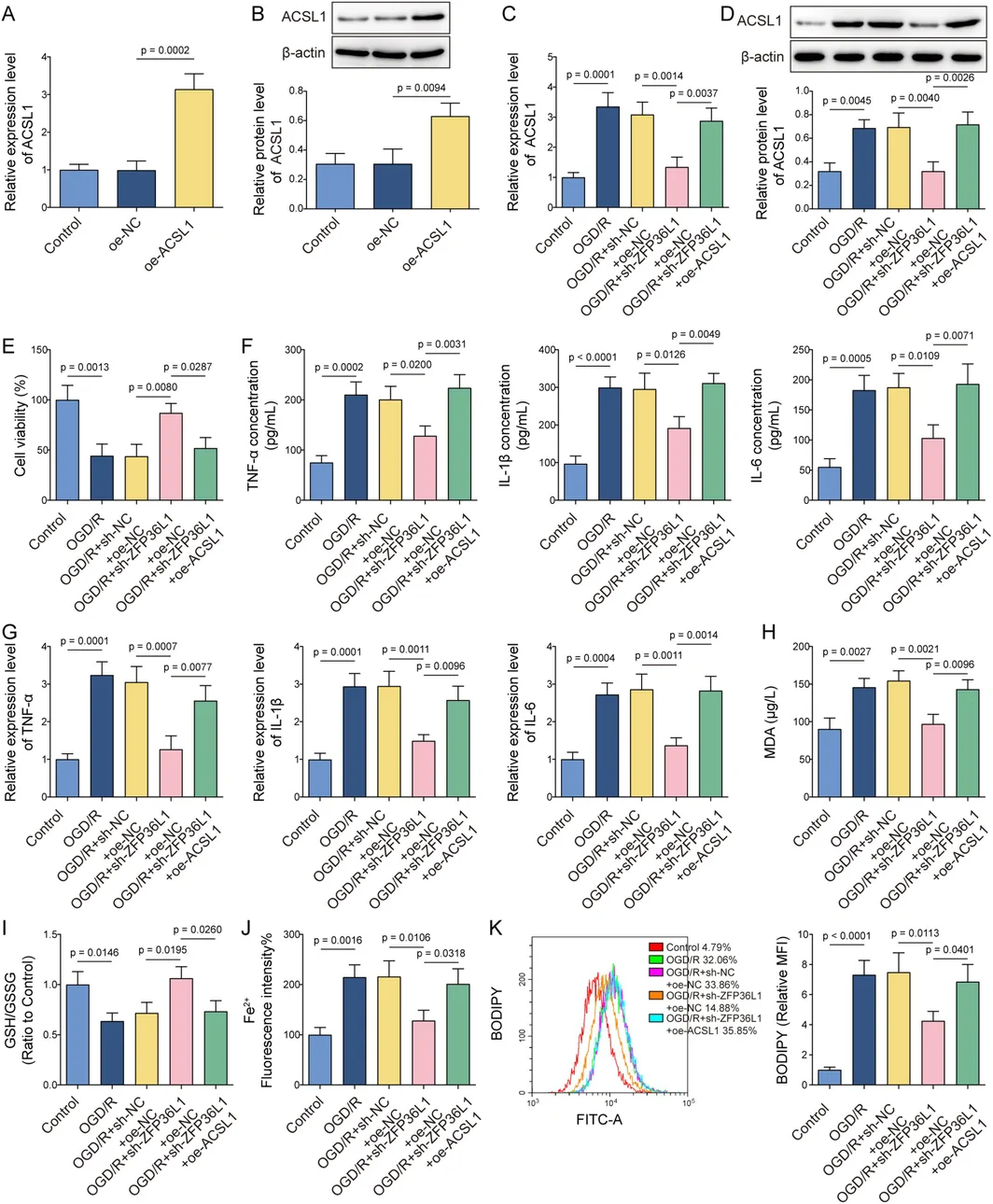
ACSL1 overexpression reversed ZFP36L1 knockdown‐mediated suppression of ferroptosis and inflammation in OGD/R‐induced microglia. BV‐2 cells were transfected with oe‐ACSL1. (A, B) ACSL1 expression was measured using RT‐qPCR and western blot. BV‐2 cells were transfected with sh‐ZFP36L1 or oe‐ACSL1, followed by OGD/R. (C, D) ACSL1 expression was measured using RT‐qPCR and western blot. (E) Cell viability was evaluated using the CCK‐8 assay. (F‐G) TNF‐*α*, IL‐1*β*, and IL‐6 levels in supernatants were determined using ELISA and RT‐qPCR. (H‐I) MDA levels and GSH/GSSG ratio were examined using the corresponding assay kits. (J) Fe^2+^ levels were measured using the FerroOrange probe. (K) Lipid peroxidation levels were measured using the C11‐BODIPY 581/591 fluorescent probe. All experiments were conducted 3 times independently, *n* = 3 per group.

### 3.7 ZFP36L1 Silencing Alleviated Cerebral Ischemic Injury in MCAO/R Mice by Decreasing ACSL1 Expression

C57BL/6 mice were used to establish an IS animal model using the MCAO/R method. Lateral ventricle injections were performed to deliver adenoviruses containing sh‐ZFP36L1 alone or in combination with oe‐ACSL1. The stereotaxic coordinates were as follows: anteroposterior +1.0 mm, mediolateral −0.59 mm; and dorsoventral −2 mm from the bregma (Figure S2). After 3 days, MCAO/R surgery was performed. TTC staining revealed extensive cerebral infarction in the MCAO/R group. However, ZFP36L1 knockdown reduced the cerebral infarction size, and ACSL1 overexpression reversed the protective effect of ZFP36L1 knockdown on infarct size (Figure 6A,B). Neurological severity scores and Nissl staining were used to assess neuronal damage. Our results showed that MCAO/R induced significant neuronal damage. ZFP36L1 knockdown largely alleviated neuronal damage, which was abolished by ACSL1 overexpression (Figure 6C,D). As presented in Figure 6E,F, ACSL1 expression was upregulated in MCAO/R mice. Notably, ZFP36L1 knockdown inhibited ACSL1 expression in MCAO/R mice, whereas ACSL1 overexpression reversed the effect of ZFP36L1 knockdown on ACSL1 expression. Additionally, TNF‐*α*, IL‐1*β*, and IL‐6 levels were significantly elevated in MCAO/R mice; however, ZFP36L1 knockdown decreased the levels of these indicators. ACSL1 overexpression further counteracted the ZFP36L1 knockdown‐mediated decrease in TNF‐*α*, IL‐1*β*, and IL‐6 levels (Figure 6G). Finally, ACSL1 overexpression reversed the ability of ZFP36L1 knockdown to alleviate the MCAO/R‐induced elevations in MDA and suppression of the GSH/GSSG ratio (Figure 6H,I). Taken together, ZFP36L1 knockdown suppressed ACSL1 expression and ameliorated brain injury in MCAO/R mice.

**FIGURE 6:**
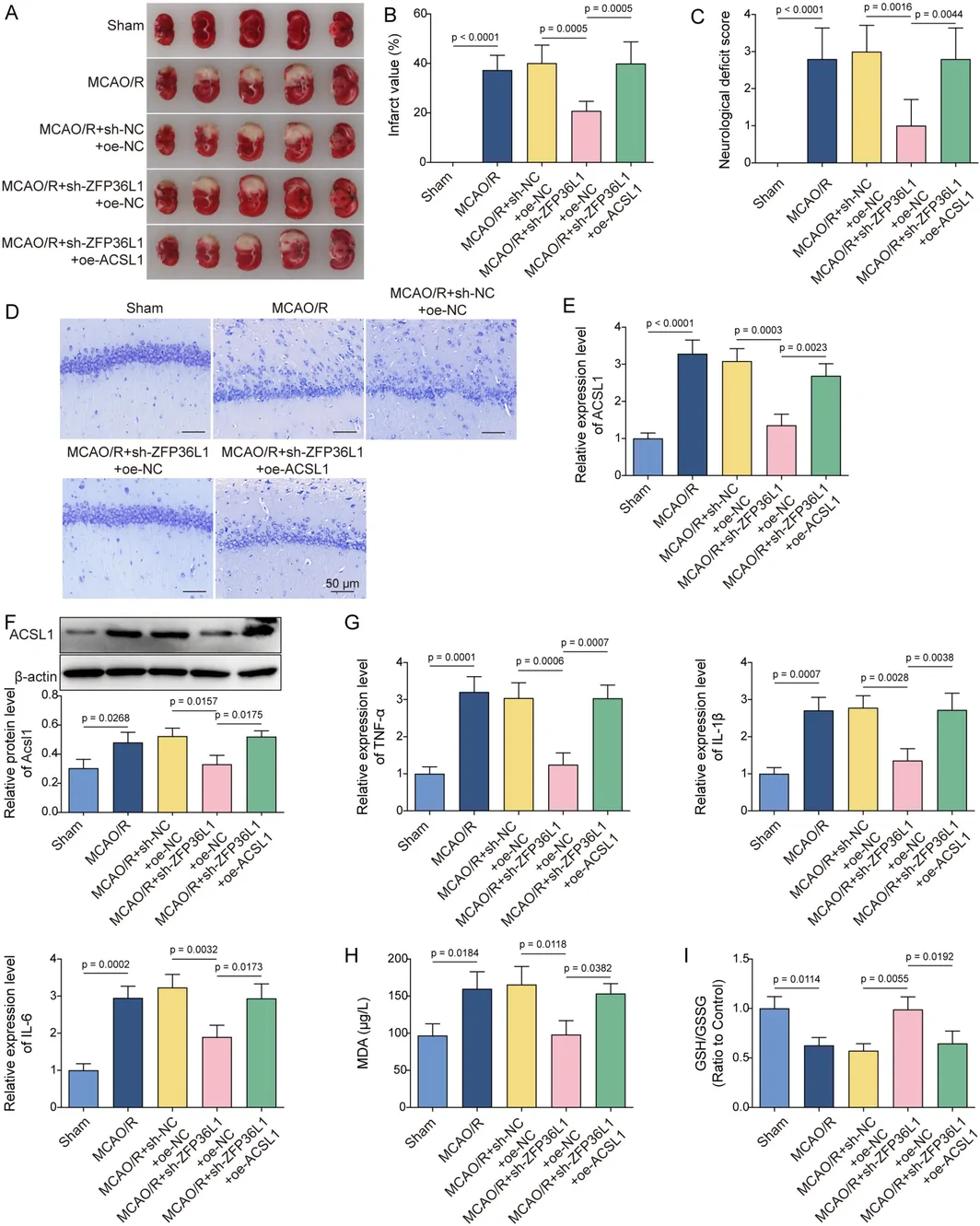
ZFP36L1 silencing alleviated cerebral ischemic injury in MCAO/R mice by decreasing ACSL1 expression. C57BL/6 mice were used to construct an IS model using the MCAO/R method, and sh‐ZFP36L1 alone or combined with oe‐ACSL1 was injected into the lateral ventricle for intervention. (A‐B) Cerebral infarct size was evaluated by TTC staining (*n* = 5). (C) Neurological deficit scores of each mouse (*n* = 5). (D) Neuronal damage in the mice was evaluated using Nissl staining (*n* = 5). (E‐F) ACSL1 expression was measured by RT‐qPCR and western blot. (G) TNF‐*α*, IL‐1*β*, and IL‐6 levels in tissues were determined using RT‐qPCR. (H‐I) MDA levels and GSH/GSSG ratios were examined using the corresponding assay kits. For E‐I, experiments were conducted 3 times independently, *n* = 3 per group.

## 4 Discussion

IS is a common cerebrovascular disease with high morbidity and mortality. Microglia are the main immune cells that defend against brain injury and play an essential and complex role in IS [21]. As previously reported, microglial ferroptosis exacerbates neuroinflammation [22]. The inhibition of ferroptosis is a novel strategy for preventing neuroinflammation [23, 24]. The mechanism underlying microglial ferroptosis in IS remains unclear. At present, the molecular mechanism of ferroptosis in microglia under IS conditions has not been fully elucidated. Here, we demonstrate that the ZFP36L1/FTO/ACSL1 axis is involved in IS progression by modulating ferroptosis and neuroinflammation in microglia, providing new directions and molecular targets for IS treatment in the future.

ACSL1, a member of the long‐chain ACSL family that participates in lipid metabolism, is implicated in various diseases, including ovarian cancer, acute myocardial infarction, and IS [20, 25, 26]. For instance, ACSL1 may participate in the immune response and ferroptosis of various immune cells, including monocytes, contributing to COVID‐19‐induced IS, and is considered a therapeutic target for COVID‐19‐induced IS [13]. More importantly, growing evidence indicates that ACSL1 is closely linked to ferroptosis [12]. Some studies showed that ACSL1, a gene associated with ferroptosis, is elevated in IS patients [26, 27]. ACSL1 also regulates inflammation in various diseases, including severe acute pancreatitis and murine coronavirus infection [12, 28]. However, the effects of ACSL1 on ferroptosis and inflammation in microglia following IS have not been fully elucidated. In this study, by mining the GSE16561 dataset, we found that ACSL1 expression was elevated in the blood samples of patients with IS, which was validated in our own collected blood samples from IS patients and OGD/R‐induced microglia. Furthermore, our findings demonstrated that ACSL1 knockdown suppressed ferroptosis and inflammation in OGD/R‐induced microglia and ameliorated IS‐related brain injury.

As a common epigenetic mechanism, m6A modification regulates gene expression by affecting the stability and translation of modified transcripts [29]. It is well known that m6A modification of RNA requires m6A‐modifying enzymes, mainly methyltransferases (METTL3, METTL14, and WTAP), demethylases (FTO and AlkB homolog 5), and m6A “reader” proteins (YTHDF1‐3), all of which can affect RNA m6A modification and thereby regulate target gene expression [30]. For example, METTL14 mediates m6A methylation of TCP1, which increases TCP1 transcript stability and its expression in acute myeloid leukemia [31]. FTO, a common demethylase that removes the m6A modification of RNA, also affects the mRNA stability of genes and their expression [32]. As previously described, FTO decreases the m6A modification of DDIT4 mRNA to enhance the mRNA stability of DDIT4 and increase DDIT4 expression, thus participating in prostate cancer [32]. Here, METTL3, METTL14, and FTO were abnormally expressed in IS patients, while only FTO had a negative relationship with ACSL1. Bioinformatics predictive analysis showed that ACSL1 had multiple methylation sites, implying that FTO probably affects ACSL1 expression by regulating m6A modification of ACSL1 mRNA. Our experiments validated that FTO decreases the m6A modification of ACSL1 mRNA to reduce the stability of ACSL1 mRNA and ACSL1 expression. Notably, FTO was reported to be downregulated in microglia after IS and to exert a protective role in IS. In our study, FTO expression was reduced in clinical samples and OGD/R‐treated microglia, similar to a published study [33].

As an adenylate‐uridylate‐rich RNA‐binding protein, ZFP36L1 can bind to the RNA of target genes to mediate RNA decay [34]. For instance, ZFP36L1 specifically binds to HIF1A, CCND1, and E2F1 to promote mRNA degradation, thereby inhibiting the expression of HIF1A, CCND1, and E2F1 [34]. In the current study, we found that FTO is a target gene of ZFP36L1. Our results revealed that ZFP36L1 interacts with FTO mRNA, further reducing its stability and lowering FTO expression in microglia. More importantly, ZFP36L1 has been widely investigated in multiple diseases and plays a crucial role in these conditions [35, 36]. In addition, a review reported that the ZFP36 family proteins participate in immune responses and inflammatory diseases [37]. Unfortunately, no evidence has yet clarified the role of ZFP36L1 in IS. In the current study, we found ZFP36L1 expression was abnormally upregulated in the blood samples of patients with IS and OGD/R‐induced microglia. Our findings further revealed that ZFP36L1 knockdown suppressed ferroptosis and inflammation in OGD/R‐induced microglia. Moreover, ZFP36L1 knockdown reduced cerebral infarction size and alleviated neuronal damage in MCAO/R‐induced mice. This evidence supports the beneficial effects of ZFP36L1 knockdown on IS. Notably, rescue experiments revealed that ACSL1 overexpression reversed the ZFP36L1 knockdown‐mediated effects in both OGD/R‐induced microglia and MCAO/R‐induced mice.

In conclusion, our results elucidate the molecular mechanisms underlying inflammation and ferroptosis in microglia after IS. Specifically, ZFP36L1 reduced the stability of FTO mRNA, decreased FTO expression, and diminished the FTO‐mediated suppression of m6A modification in ACSL1 mRNA, leading to elevated ACSL1 expression. This, in turn, promoted ferroptosis and inflammation in OGD/R‐treated microglia and increased cerebral infarction size and neuronal damage in MCAO/R‐induced mice. These findings provide a novel mechanism for microglial ferroptosis after IS and may aid in understanding IS pathology and developing effective therapeutic strategies. However, our study has some limitations. First, we primarily focused on ferroptosis, a specific form of programmed cell death, and did not systematically evaluate the contributions of other cell death modalities, such as apoptosis, necroptosis, or autophagy, to OGD/R‐induced microglial death or ischemic brain injury. In the complex pathological environment of IS, multiple cell death pathways are often simultaneously activated and may interact. Therefore, the precise role of ferroptosis in the overall microglial loss and subsequent neuroinflammation remains to be fully determined. Future studies should aim to concurrently assess markers of multiple cell death pathways and use specific pharmacological inhibitors (e.g., Z‐VAD‐FMK for apoptosis, Necrostatin‐1 for necroptosis) alongside ferroptosis inhibitors (e.g., Ferrostatin‐1) to determine the relative contributions and potential crosstalk of these mechanisms in IS. In addition, although weighted analysis confirmed that the statistical power of the sample size in this study exceeded 80%, meeting the basic experimental requirements, larger sample sizes would further improve the reliability of the results. Consequently, large‐scale studies are needed to validate and extend the findings of this work.

## Funding

This work was supported by ZFP36L1 exacerbates ferroptosis of microglia and neuroinflammation in ischemic stroke by enhancing the epigenetic modification of ACSL1 through transcriptional regulation (H2025405076).

## Ethics Statement

This study was approved by the Medical Ethics Committee of the First Affiliated Hospital of Hebei North University (K202418P). All participants signed informed consent forms.

The animal experiments were approved by the Committee of Experimental Animals of Hebei North University (HBNU20240822122).

## Conflicts of Interest

The authors declare no conflicts of interest.

## Supporting information


**Figure S1:** ACSL1 was highly expressed in blood samples of IS patients and OGD/R‐induced microglia, and ferroptosis was promoted in IS (A) Differentially expressed genes of RNAs from the GSE16561 dataset were presented as a volcano plot. (B) The function of differentially expressed genes was analyzed using GO functional enrichment analysis. (C) TNF‐*α*, IL‐1*β*, and IL‐6 levels in clinical samples were detected using ELISA. BV‐2 cells were treated with OGD/R exposure. (D) Cell viability was evaluated using CCK‐8. (E) TNF‐*α*, IL‐1*β*, and IL‐6 levels in supernatants were determined using ELISA. (F) 10 highly expressed genes from the GSE16561 dataset were shown as a heat map. (G) GSEA plot showing the relationship of differentially expressed genes with ferroptosis pathway in the GSE16561 dataset. (H) ACSL1 expression in clinical samples was assessed using RT‐qPCR (Healthy: *n* = 15, Stroke: *n* = 20). (I) MDA level and GSH in clinical samples were examined using the corresponding assay kits. BV‐2 cells were treated with OGD/R exposure. (J‐K) ACSL1 expression was measured using RT‐qPCR and western blot. (L‐M) MDA level and GSH/GSSG ratio were examined using corresponding assay kits. (*N*) The Fe^2+^ level was measured using the FerroOrange probe. For C‐E and I‐N, experiments were conducted 3 times independently, *n* = 3 per group.

**Figure S2:** Trace diagram of lateral ventricle injection.
The injection needle was inserted into the lateral ventricle to deliver adenoviruses containing sh‐ZFP36L1, oe‐ACSL1 or their negative control plasmids into brain. Four days later, brain tissue was taken for HE staining and the injection marks were observed (The arrow indicates needle marks).

## Data Availability

The data that support the findings of this study are available from the corresponding author upon reasonable request.
