# Supplementary figures and images for: ZFP36L1 Enhances Microglial Ferroptosis in Ischemic Stroke by Reducing FTO‐Mediated N6‐Methyladenosine Demethylation of ACSL1 mRNA
Source: Kaohsiung J Med Sci. 2026 Apr 29:e70212. Online ahead of print. doi: 10.1002/kjm2.70212 (PMC13399683; doi:10.1002/kjm2.70212)

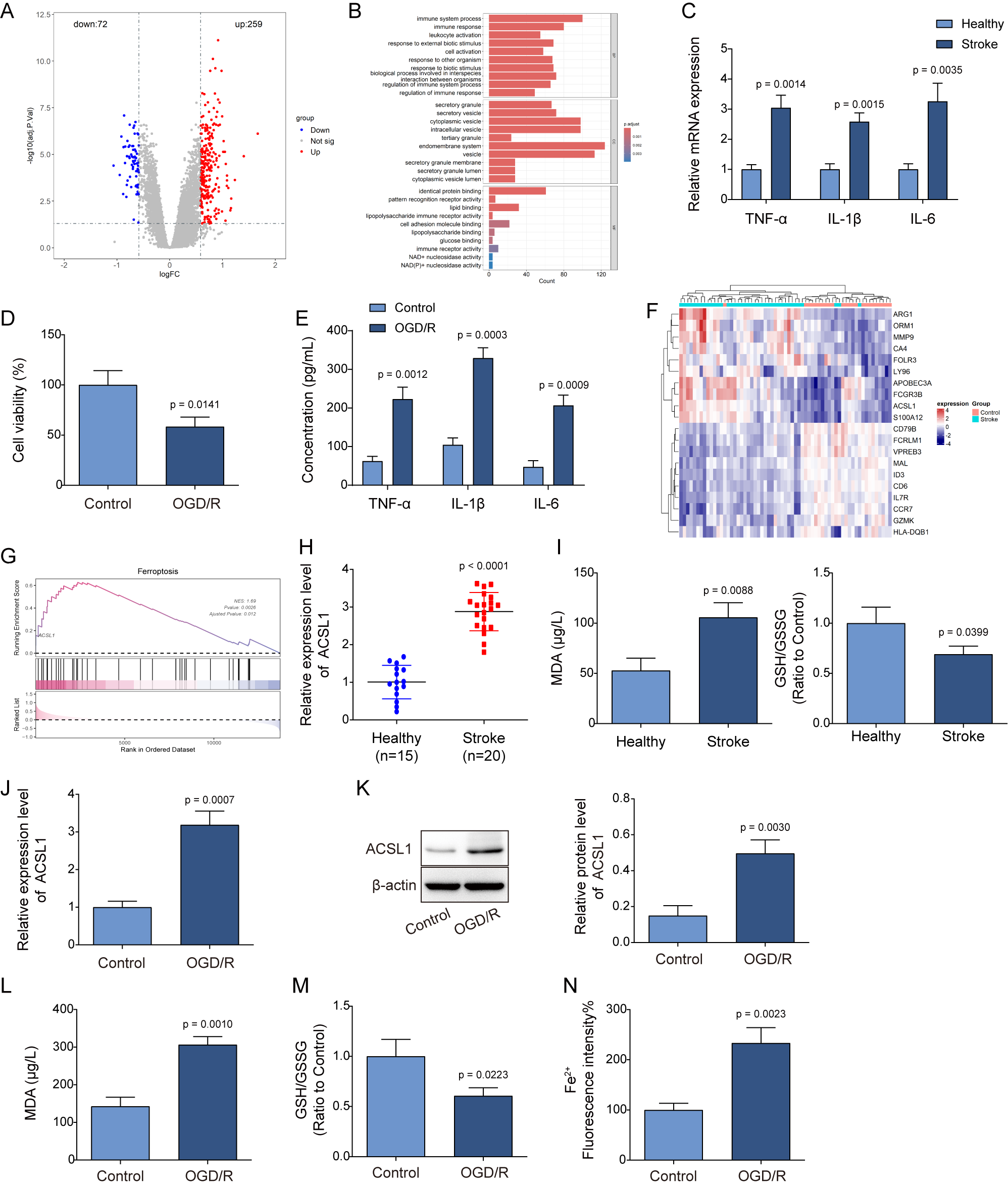

Supplement: Supplementary file 1 — Figure S1: ACSL1 was highly expressed in blood samples of IS patients and OGD/R‐induced microglia, and ferroptosis was promoted in IS (A) Differentially expressed genes of RNAs from the GSE16561 dataset were presented as a volcano plot. (B) The function of differentially expressed genes was analyzed using GO functional enrichment analysis. (C) TNF‐α, IL‐1β, and IL‐6 levels in clinical samples were detected using ELISA. BV‐2 cells were treated with OGD/R exposure. (D) Cell viability was evaluated using CCK‐8. (E) TNF‐α, IL‐1β, and IL‐6 levels in supernatants were determined using ELISA. (F) 10 highly expressed genes from the GSE16561 dataset were shown as a heat map. (G) GSEA plot showing the relationship of differentially expressed genes with ferroptosis pathway in the GSE16561 dataset. (H) ACSL1 expression in clinical samples was assessed using RT‐qPCR (Healthy: n = 15, Stroke: n = 20). (I) MDA level and GSH in clinical samples were examined using the corresponding assay kits. BV‐2 cells were treated with OGD/R exposure. (J‐K) ACSL1 expression was measured using RT‐qPCR and western blot. (L‐M) MDA level and GSH/GSSG ratio were examined using corresponding assay kits. (N) The Fe2+ level was measured using the FerroOrange probe. For C‐E and I‐N, experiments were conducted 3 times independently, n = 3 per group. [file KJM2-9999-e70212-s002.tif]

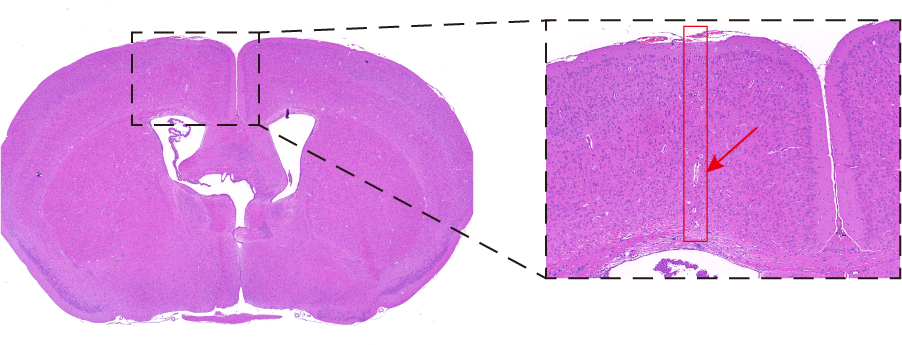

Supplement: Supplementary file 2 — Figure S2: Trace diagram of lateral ventricle injection. The injection needle was inserted into the lateral ventricle to deliver adenoviruses containing sh‐ZFP36L1, oe‐ACSL1 or their negative control plasmids into brain. Four days later, brain tissue was taken for HE staining and the injection marks were observed (The arrow indicates needle marks). [file KJM2-9999-e70212-s001.tif]
